# Linear structural features of Wilson’s disease and its correlation with neurological symptoms

**DOI:** 10.1097/MD.0000000000031386

**Published:** 2022-12-16

**Authors:** Sheng-Peng Diao, Chun-Xiao Lǚ, Ye-Qing Huang, Zhi-Hua Zhou, Ai-Qun Liu, Ming-Fan Hong

**Affiliations:** a Department of Neurology, The First Affiliated Hospital, Jinan University, Guangzhou, China; b Department of Neurology, College of Clinical Medicine, The First Affiliated Hospital, Guangdong Pharmaceutical University, Guangzhou, China.

**Keywords:** Burke Fahn Marsden Movement subscale (BFM-M), linear brain measurement, magnetic resonance imaging (MRI), neurological symptoms, Wilson’s disease (WD)

## Abstract

To measure the linear structure of the brain in patients with Wilson’s disease (WD) and analyze its correlation with neurological symptoms. A total of 174 patients diagnosed with WD were enrolled. According to the type of clinical presentation, the patients with WD were divided into two groups: neurological (NWD) and hepatic (HWD). Sixty healthy volunteers were assigned to a control group. All patients with WD and healthy controls underwent brain magnetic resonance imaging (MRI). The severity of the neurological symptoms was assessed using the Burke Fahn Marsden Movement subscale (BFM-M). Linear brain measurements were performed using T1-weighted MRI scans of all the patients, and the correlation between these linear indices and BFM-M score was investigated. The Huckman index, third ventricle width, and sulcus width of the NWD group were significantly higher than those of the HWD and control groups (*P* < .05). The frontal horn index, ventricular index, and lateral ventricular body width index of the NWD group were significantly lower than those of the HWD and control groups (*P* < .05). The Huckman index and third ventricle width of the HWD group were higher than those of the control group (*P* < .05), whereas the body width index of the lateral ventricle was lower than that of the control group (*P* < .05). The BFM-M score correlated with the Huckman index (*r* = 0.29, *P* < .05), third ventricle width (*r* = 0.426, *P* < .001), and lateral ventricular body width index (*r* = −0.19, *P* < .05). This study demonstrated significant changes in the linear structure of patients with WD. Linear brain measurement analysis could be used as a potential method to assess the severity of neurological symptoms in WD.

## 1. Introduction

Wilson’s disease (WD) is a rare autosomal recessive disorder of copper metabolism caused by ATP7B gene mutations on chromosome13 (13q14.3) ATP7B gene encodes a copper-transporting P-type ATPase. The dysfunction of ATP7B causes excessive copper accumulation in the liver, brain, kidneys, and other organs. Liver disease and neurological symptoms are WD’s most common clinical features.^[1–3]^ Copper deposits in the brain gradually damage the neurons, leading to neurological symptoms.

Magnetic resonance imaging (MRI) is the most commonly used imaging method for evaluating brain lesions in WD. The most common brain MRI findings are symmetrical or asymmetric hyperintensities in T2-weighted images of the deep gray matter nuclei and brain stem.^[4]^ In recent years, many studies have been conducted using brain MRI in patients with WD. Brain MRI is often used for the diagnosis of WD,^[5]^ treatment monitoring,^[6]^ and assessment of the severity of WD disease.^[6,7]^ Recently, a semiquantitative brain MRI scale has been proposed, and the correlations between the Unified Wilson’s Disease Rating Scale and brain MRI were etsabilshed.^[7]^ However, most studies have focused on the relationship between the location of brain damage and neurological symptoms, and no unified understanding has been established.

Copper widely accumulates in the brain of patients with WD, causing chronic damage to the brain parenchyma, resulting in morphological changes and varying degrees of brain atrophy.^[8–10]^ Brain atrophy has been assessed by software analysis of brain MRI and has found that the severity of brain atrophy correlates with functional and neurological impairment in patients with Wilson disease.^[11]^ Atrophy was also involved in brain MRI semiquantitative scale to assess the radiological severity of WD.^[6,12]^ Although there is a close relationship between brain atrophy and neurological symptoms in patients with WD, there are very few studies in this area, and the method of evaluating brain atrophy by software analysis of brain MRI is complex and difficult to grasp by clinicians. A simple and quick assessment method for brain atrophy will significantly help clinicians study WD brain MR imaging and neurological symptoms. Linear brain measurement is an essential method for assessing morphological changes and atrophy of the brain.^[13–15]^ No studies have been conducted on the relationship between linear WD brain measurements and neurological symptoms. This study aimed to collect linear measurement data from WD brain MRI, assess the severity of neurological symptoms, and evaluate their relationship.

## 2. Objects and methods

### 2.1. Research object

We collected 174 WD patients admitted to the First Affiliated Hospital of Guangdong Pharmaceutical University from January 2016 to December 2021, including 93 males and 81 females aged 11 to 62 years (mean age 21.61 ± 9.21 years). All cases met the diagnostic criteria of WD,^[3]^ ceruloplasmin < 0.2 g/L, 24h urine copper > 100 μg, corneal Kayser-Fleischer (K-F) ring positivity, and a positive family history. Sixty well-matched healthy controls were enrolled in the study. All subjects signed an informed consent form that was approved by the hospital ethics committee.

Patients with WD were classified according to their clinical presentation.^[3]^ The 114 patients with neurological signs were classified into the NWD group, and the 60 patients with hepatic symptoms and without neurological were assigned to the HWD group.

### 2.2. Brain-linearity measurement

All patients with WD and healthy controls were scanned using 3.0T GE silent MR, conventional axial T1-weighted [repetition time (TR)/echo time (TE) = 3600 ms/100 ms], a layer thickness of 5.0 mm, and a layer spacing of 0.5 mm were used. The measurement indices of each imaging data were measured, recorded, and sorted by two radiologists in professional imaging doctors using computers.

**Figure 1.**
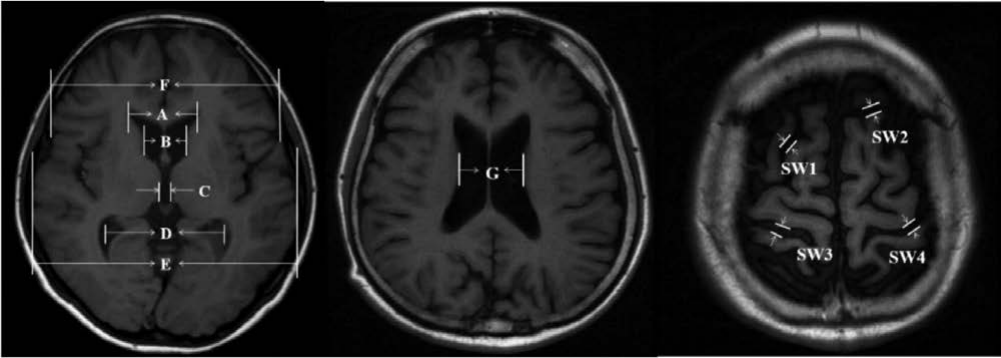
Linear measurements in the brain T1-weighted MRI. A = frontal horns greatest distance, B = minimum distance of caudate nucleus head, C = widest transverse diameter of the third ventricle, D = the choroid plexuses distance, E = maximum internal transverse diameter of skull, F = frontal bones’ greatest external distance, G = maximum external diameter between the lateral ventricular bodies, SW = the four widest sulci at the skull vault.

Linear brain measurements were performed using T1-weighted MRI scans of all the patients. The primary measured linear indices^[16–19]^ were calculated using the formulae presented in Table 1 and Figure 1.

**Table 1:** Description of radiological linear indices and ratios.

Indices & ratios	Calculation method	Formula^*^
Huckman index	Sum of the frontal horns greatest distance and the minimum distance of caudate nucleus head	A + B
Third ventricle width	The widest transverse diameter of the third ventricle	C
Ventricular index	The choroid plexuses distance divided by the frontal horns greatest distance	D/A
Lateral ventricular body width index	Maximum internal transverse diameter divided by the maximum external diameter between the lateral ventricular bodies	E/G
Frontal horn index	Frontal bones’ greatest external distance divided by the frontal horns greatest distance	F/A
Sulcus width	The average of the four widest sulci at the skull vault	SW

* Refer to Figure 1.

### 2.3. Burke Fahn Marsden Movement subscale (BFM-M)^[20]^ evaluation

In this study, the severity of neurological symptoms was assessed by two neurologists by consensus using the BFM-M. The higher the score, the more serious the corresponding neurological symptoms, with a maximum score of 120 points.

### 2.4. Statistical methods

Spss19.0 statistical software was used to process the data. The data were tested for homogeneity of variance, and data conforming to a normal distribution were measured as the mean ± standard deviation (x±s). One-way analysis of variance (ANOVA) was used to compare multiple groups. When the conflict was homogeneous, the least significant difference (LSD) method was used for pairwise comparisons between multiple groups. The Howell method is used when the variance is uneven; Pearson correlation analysis analyzes the correlation between each index and BFM-M score. The test level was α = 0.05 and *P* < .05, indicating that the difference was statistically significant.

## 3. Results

### 3.1. Clinical data of WD patients and healthy controls

There were significant differences in serum copper, urinary copper, and ceruloplasmin levels between patients with WD and healthy controls (*P* < .05). However, there were no significant differences in other indices, as shown in Table 2.

**Table 2:** Comparison of basic conditions of WD patients and healthy controls.

Variables	NWD group (N = 114)	HWD group (N = 60)	Healthy control group (N = 60)
Male/female	62/52	31/29	36/24
Age (yr)	23.92 ± 8.3	19.78 ± 9.89	21.42 ± 9.81
Disease course (yr)	6.21 ± 2.72	5.98 ± 2.28	/
Serum copper (μmol/L)	0.39 ± 0.08^*^	0.38 ± 0.06^*^	0.87 ± 0.09
Urine copper (μg/24h)	325 ± 78^*^	405 ± 148^*^	62 ± 21
Ceruloplasmin (μg/mL)	114.85 ± 18.21^*^	117.65 ± 16.92^*^	362.35 ± 38.64
BFM-M score	26.07 ± 17.55	0	0

Value in mean ± SD.

BFM-M = Burke Fahn Marsden Movement subscale, HWD = hepatic, NWD = neurological, WD = Wilson’s disease.

* Compared with the healthy control group, *P* < .05.

### 3.2. Characteristics of linear brain structure in WD patients

The linear brain measurement indices included the Huckman index, third ventricle width, ventricular index, lateral ventricular body width index, frontal horn index, and sulcus width. The Huckman index, third ventricle width, and sulcus width were significantly higher in the NWD group than those in the HWD and control groups (*P* < .05). The frontal horn index, ventricular index, and lateral ventricular body width index of the NWD group were significantly lower than those of the HWD and control groups (*P* < .05). In the comparison between the HWD and control groups, the Huckman index and third ventricle width in the HWD group were higher than those in the control group (*P* < .05), whereas the lateral ventricular body width index of the lateral ventricle was lower than that in the control group (*P* < .05), Table 3.

**Table 3:** Comparison of MRI linear measurement indexes in NWD, HWD, and healthy control groups.

Variables	Huckman index	Frontal horn index	Ventricular index	Third ventricle width (mm)	Lateral ventricular body width index	Sulcus width
NWD (N = 114)	54.38 ± 6.5^*^^,^^†^	3.83 ± 0.3^*^^,^^†^	1.66 ± 0.17^*^^,^^†^	9.1 ± 2.73^*^^,^^†^	4.64 ± 0.67^*^^,^^†^	2.91 ± 1.1^*^^,^^†^
HWD (N = 60)	47.24 ± 5.56^‡^	4.01 ± 0.31	1.74 ± 0.17	5.86 ± 2.47^‡^	5.69 ± 1.07^‡^	2.11 ± 1.0^‡^
Healthy control group (N = 60)	43.48 ± 2.59	4.14 ± 0.24	1.75 ± 0.12	4.14 ± 1.19	6.78 ± 3.13	0.69 ± 0.78

Value in mean ± SD.

HWD = hepatic, MRI = magnetic resonance imaging, NWD = neurological.

* Compared with NWD and HWD, *P* < .05.

† Compared with NWD and healthy control, *P* < .05.

‡ Compared with the HWD and healthy control *P* < .05.

### 3.3. Relationship between the linear brain measurements and BFM-M score

The BFM-M score was positively correlated with the Huckman index and third ventricle width (*r* = 0.29, *P* < .05; *r* = 0.426, *P* < .001) and negatively correlated with the lateral ventricular body width index (*r* = −0.19, *P* < .05). The BFM-M scores were not correlated with the frontal horn index, ventricular index, or sulcus width, Table 4.

**Table 4:** Correlation test results of the MRI brain linear measurement indexes and BFM-M scale in 114 WD patients with neurological symptoms.

Index	*r*	*P* value
Huckman index	0.29	.002
Frontal horn index	−0.027	.772
Ventricular index	−0.095	.313
Third ventricle width	0.426	.000
Lateral ventricular body width index	−0.19	.043
Sulcus width	−0.072	.455

BFM-M = Burke Fahn Marsden Movement subscale, MRI = magnetic resonance imaging, WD = Wilson’s disease.

## 4. Discussion

WD is a disorder of copper metabolism disorder. The clinical symptoms are related to the deposition of copper in different organs and tubes. Copper deposits in the brain gradually damage the neurons, leading to neurological symptoms. In recent years, the relationship between neurological symptoms and brain imaging in WD patients has become a research hotspot.

**Figure 2.**
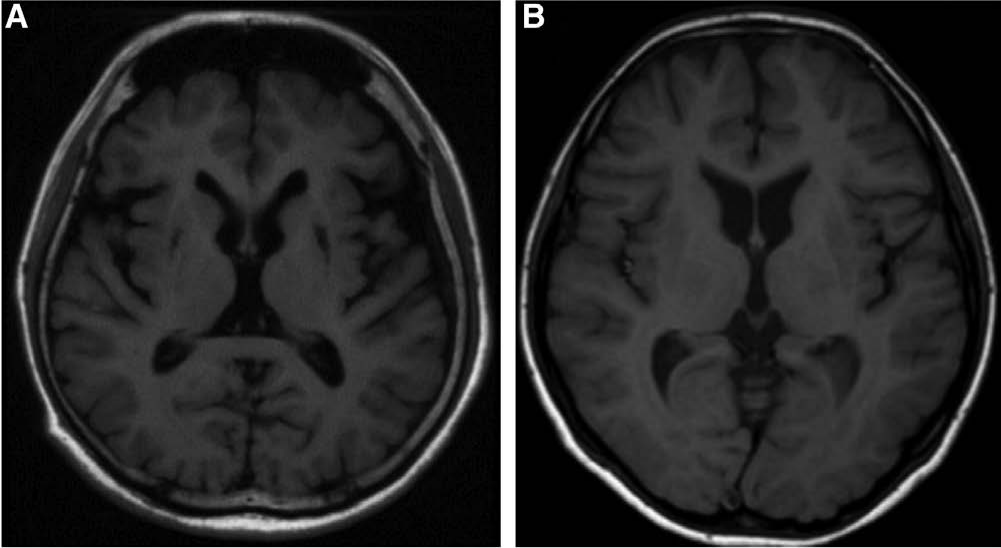
T1WI imaging of some special patients. A, T1WI imaging of typical NWD patients, with obvious symmetrical abnormal signal in the basal ganglia; B, T1WI imaging of a few NWD patients, with no obvious abnormal signal in the basal ganglia, but obvious changes in the linear structure of the ventricle. WD = Wilson’s disease.

There have been many studies on the relationship between brain MRI and neurological symptoms in patients with WD; however, there is no unified understanding.^[21–26]^ Some studies have suggested that tremor is related to lesions of the putamen nucleus, lateral thalamic nucleus, and substantia nigra; high muscle tone and bradykinesia are related to putamen lesions; dysarthria is related to the putamen and caudate nucleus lesions; ataxia is related to the cerebellum, basal pontine and thalamus lesions; and chorea is related to caudate nucleus lesions.^[21,22]^ However, some scholars believe that there is no correlation between brain MRI and neurological symptoms in patients with WD and reported that 10.5% of patients with WD have neurological symptoms without abnormal brain MRI findings.^[23]^ In this study, 9.65% of patients with NWD had neurological symptoms but no abnormal MRI lesions, as shown in Figure 2. We speculate that patients with WD may have unknown brain MRI features related to neurological symptoms.

Abnormal deposition of copper in the brain tissue of patients with WD causes cerebral ischemia, edema, neuronal degeneration, gliosis, and cystic neuronal changes. Neuronal necrosis and loss can cause morphological changes in the WD brain.^[8]^ Strecker et al^[9]^ reported that changes in brain atrophy most likely reflect early and irreversible neurodegeneration induced by copper. The incidence of brain atrophy in patients with WD is high, with a reported incidence of 88.9%.^[10]^ The incidence of brain atrophy in patients with neurological symptoms is higher than that in patients without neurological symptoms.^[27]^ Brain atrophy is closely related to neurological symptoms, and linear brain measurements can effectively reflect the degree of brain atrophy. However, there has been no research on the correlation between linear WD brain MRI measurements and neurological symptoms. In this study, brain MRI was used to measure the linear brain measurements of WD patients, which reflect the degree of brain atrophy and ventricular dilation, including the Huckman index, third ventricle width, ventricular index, lateral ventricular body width index, frontal horn index and sulcus width,^[13–15]^ and to study the relationship between the linear brain measurements and neurological symptoms.

The sulcus width reflects cortical brain atrophy, while the third ventricle width, frontal horn index, ventricular index, and lateral ventricular body width index reflect the atrophy of deep hemispheric structures.^[14,28]^ The analysis of the linearly measured indices in this study showed that the Huckman index, third ventricle width, and sulcus width in the NWD group were significantly higher than those in the HWD and the control groups. The frontal horn, ventricular, and lateral ventricular body width index in the NWD group were significantly lower than those in the HWD and control groups. The above results show that the linear measurement changes in cortical and subcortical structures in patients with neurological symptoms are more pronounced, suggesting that the damage caused by copper in patients with neurological symptoms is damage to the whole brain.

When the HWD and control groups were compared, significant differences were observed in the Huckman index, lateral ventricular body width index, and third ventricle width. However, these differences were not as significant as those between the NWD and the control groups. Linear brain measurements changed significantly in the HWD group, resulting in brain morphological changes in WD patients, suggesting that copper damage to the brain in the HWD group reached a particular stage. However, this damage did not lead to neurological symptoms. We must be alert to neurological symptoms in patients with HWD with significant changes in linear brain measurements.

**Figure 3.**
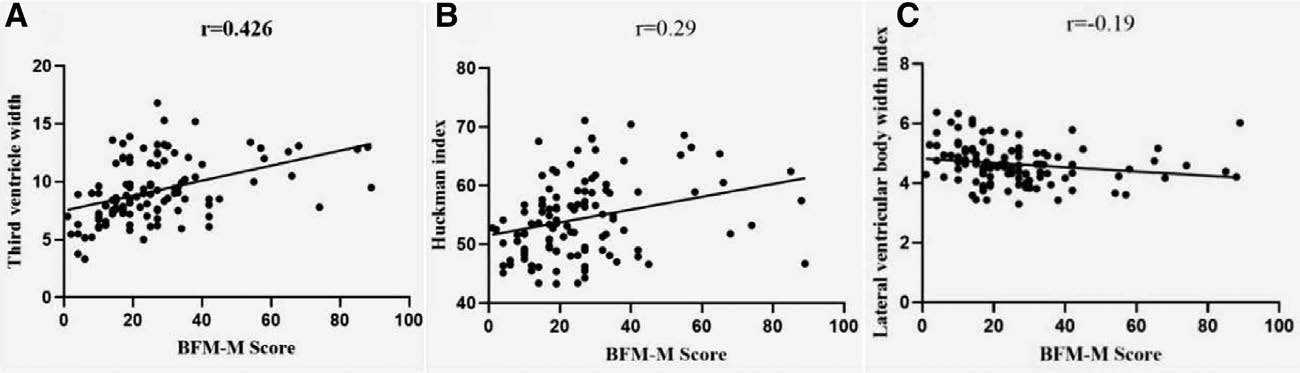
Regression curve showing the relationship between the BFM-M score and linear structure. A, BFM-M score correlated with third ventricle width (*r* = 0.426, *P* < .001); B, BFM-M score correlated with the Huckman index (*r* = 0.29, *P* < .05); C, BFM-M score correlated with the lateral ventricular body width index (*r* = −0.19, *P* < .05). BFM-M = Burke Fahn Marsden Movement subscale.

Dystonia is the most important clinical manifestation of neurological symptoms in WD.^[2]^ We used the BFM-M to evaluate the severity of dystonia in patients with WD and studied its correlation with linear brain measurements. The results showed that the BFM-M score was positively correlated with the Huckman index and the third ventricle width. In contrast, the BFM-M score was negatively correlated with the lateral ventricular body width index. Among the indices, the correlation between the BFM-M score and third ventricle width was the best (*r* = 0.426, *P* < .001), followed by the Huckman index (*r* = 0.29, *P* < .01), and lateral ventricular body width index (*r* = −0.19, *P* < .05), as shown in Figure 3. The basal ganglia, particularly the lenticular nucleus, is the core area of brain lesions in patients with WD. The involvement of the basal ganglia often causes neurological symptoms. The third ventricle width is an important indicator of brain atrophy in the basal ganglia.^[14,28]^ Third ventricle width had the best correlation with the BFM-M score, suggesting that it is important to evaluate brain degeneration and predict neurological symptoms in WD.

In linear brain measurements, the Huckman index, third ventricle width, and lateral ventricular body width index were correlated with the severity of neurological symptoms. Therefore, patients with WD should be closely monitored. Once the linear brain measurement of patients with WD changes, possible factors leading to the occurrence or aggravation of neurological symptoms should be screened. The following measures can be taken: taking drugs regularly, strengthening the treatment for expelling copper, and protecting the brain cells to avoid the occurrence or aggravation of neurological symptoms.

This study had some limitations. First, this was a single-center retrospective study, and the data might have been limited by referral bias. Therefore, future research may consider employing multicenter longitudinal designs. Second, linear measurements cannot assess the volume of brain atrophy, but this method does not require software. Despite these limitations, the current study used a much simpler technique (visualizations and diameters measurements), had strong operability, and provided a new theoretical basis for studying WD imaging and neurological symptoms. We propose that linear brain measurement could be a useful method for assessing the severity of neurological WD.

## 5. Conclusion

This study demonstrated that linear brain measurements significantly changed in patients with WD and were more obvious in patients with NWD. There is a close relationship between linear brain measurements and the severity of neurological symptoms in patients with NWD. The third ventricle width, Huckman number, and lateral ventricular body width index were correlated with the neurological severity. Linear brain measurement is an available method for assessing the severity of neurological WD.

## Acknowledgments

We would like to acknowledge the subject who participants of this study.

## Author contributions

**Conceptualization:** MingFan Hong.

**Data curation:** Shengpeng Diao.

**Investigation:** AiQun Liu.

**Methodology:** ChunXiao Lǚ.

**Resources:** ZhiHua Zhou.

**Validation:** YeQing Huang.

**Writing – original draft:** ShengPeng Diao.

**Writing – review & editing:** MingFan Hong.

## References

[1] Aggarwal A Bhatt M. Wilson disease. Curr Opin Neurol. 2020;33:534–42. 32657896 10.1097/WCO.0000000000000837

[2] Członkowska A Litwin T Dusek P. Wilson disease. Nat Rev Dis Primers. 2018;4:21. 30190489 10.1038/s41572-018-0018-3 PMC6416051

[3] Chinese Society of Neurogenetics. Chinese guidelines for diagnosis and treatment of Wilson’s disease 2021. Chin J Neurol. 2021;54:310–9.

[4] Sinha S Taly AB Ravishankar S. Wilson’s disease: cranial MRI observations and clinical correlation. Neuroradiology. 2006;48:613–21. 16752136 10.1007/s00234-006-0101-4

[5] Ferenci P Caca K Loudianos G. Diagnosis and phenotypic classification of Wilson disease. Liver Int. 2003;23:139–42. 12955875 10.1034/j.1600-0676.2003.00824.x

[6] Dusek P Smolinski L Redzia-Ogrodnik B. Semiquantitative scale for assessing brain MRI abnormalities in Wilson disease: a validation study. Mov Disord. 2020;35:994–1001. 32181965 10.1002/mds.28018

[7] Rędzia-Ogrodnik B Członkowska A Bembenek J. Brain magnetic resonance imaging and severity of neurological disease in Wilson’s disease-the neuroradiological correlations. Neurol Sci. 2022;43:4405–12. 35275318 10.1007/s10072-022-06001-2

[8] Meenakshi-Sundaram S Mahadevan A Taly AB Arunodaya GR Swamy HS Shankar SK. Wilson’s disease: a clinical-neuropathological autopsy study. J Clin Neurosci. 2008;15:409–17. 18242093 10.1016/j.jocn.2006.07.017

[9] Strecker K Schneider JP Barthel H. Profound midbrain atrophy in patients with Wilson’s disease and neurological symptoms? J Neurol. 2006;253:1024–9. 16607473 10.1007/s00415-006-0151-x

[10] Fritzsch D Reiss-Zimmermann M Trampel R Turner R Hoffmann K-T Schäfer A. Seven-tesla magnetic resonance imaging in Wilson disease using quantitative susceptibility mapping for measurement of copper accumulation. Invest Radiol. 2014;49:299–306. 24220252 10.1097/RLI.0000000000000010

[11] Smolinski L Litwin T Redzia-Ogrodnik B Dziezyc K Kurkowska-Jastrzebska I Czlonkowska A. Brain volume is related to neurological impairment and to copper overload in Wilson’s disease. Neurol Sci. 2019;40:2089–95. 31147855 10.1007/s10072-019-03942-z PMC6745045

[12] Ziemssen T Akgun K Członkowska A. Serum neurofilament light chain as a biomarker of brain injury in Wilson’s disease: clinical and neuroradiological correlations. Mov Disord. 2022;37:1074–9. 35114010 10.1002/mds.28946

[13] Sachdev P Cathcart S Shnier R Wen W Brodaty H. Reliability and validity of ratings of signal hyperintensities on MRI by visual inspection and computerised measurement. Psychiatry Res. 1999;92:103–15. 10674364 10.1016/s0925-4927(99)00036-0

[14] Chrzan R Gleń A Bryll A. Computed tomography assessment of brain atrophy in centenarians. Int J Environ Res Public Health. 2019;16:3659. 31569457 10.3390/ijerph16193659 PMC6801833

[15] Hamed MA Ponce FA Lambert M. Subcortical atrophy and motor outcomes in Pallidal Deep Brain stimulation for parkinson disease. World Neurosurg. 2020;142:89–94. 10.1016/j.wneu.2020.06.046 32540287

[16] Jernigan TL Press GA Hesselink JR. Methods for measuring brain morphologic features on magnetic resonance images. Validation and normal aging. Arch Neurol. 1990;47:27–32. 2294890 10.1001/archneur.1990.00530010035015

[17] Zivadinov R Bakshi R. Role of MRI in multiple sclerosis II: brain and spinal cord atrophy. Front Biosci. 2004;9:647–64. 14766398 10.2741/1262

[18] Pelletier D Garrison K Henry R. Measurement of whole-brain atrophy in multiple sclerosis. J Neuroimaging. 2004;14(3 Suppl):11S–9S. 15228755 10.1177/1051228404266264

[19] Han Q Cheng K Zhong H. Linear measurements of healthy adults’ coronal section of hippocampus on brain magnetic resonance imaging. J Craniofac Surg. 2013;24:197–9. 23348285 10.1097/SCS.0b013e3182646ad8

[20] Krystkowiak P DuMontcel ST Vercueil L. Reliability of the Burke-Fahn-Marsden scale in a multicenter trial for dystonia. Mov Disord. 2007;22:685–89. 17274034 10.1002/mds.21392

[21] Skowrońska M Litwin T Dzieżyc K Wierzchowska A Członkowska A. Does brain degeneration in Wilson disease involve not only copper but also iron accumulation? Neurol Neurochir Pol. 2013;47:542–6. 24374999 10.5114/ninp.2013.39071

[22] Park NH Kim HS Yi SY Min BC. Multiple osteochondritis dissecans of knee joint in a patient with Wilson disease, focusing on magnetic resonance findings. Knee Surg Relat Res. 2013;25:225–9. 24369002 10.5792/ksrr.2013.25.4.225 PMC3867617

[23] Ferenci P Litwin T Seniow J Czlonkowska A. Encephalopathy in Wilson disease: copper toxicity or liver failure? J Clin Exp Hepatol. 2015;5(Suppl 1):S88–95. 26041965 10.1016/j.jceh.2014.09.002 PMC4442862

[24] Verma R Holla VV Pandey S Rizvi I. Multifocal myoclonus as a heralding manifestation of Wilson disease. J Pediatr Neurosci. 2016;11:358–60. 28217166 10.4103/1817-1745.199468 PMC5314857

[25] Dohan A Vargas O Dautry R. MR imaging features of focal liver lesions in Wilson disease. Abdom Radiol (NY). 2016;41:1811–24. 27116011 10.1007/s00261-016-0744-5

[26] Vargas O Faraoun SA Dautry R. MR imaging features of liver involvement by Wilson disease in adult patients. Radiol Med. 2016;121:546–56. 27105862 10.1007/s11547-016-0635-4

[27] Batool Hamdani SS Cheema HA Saeed A Malik HS Sehar T. Electrocardiographic manifestations in paediatric Wilson Disease. J Ayub Med Coll Abbottabad. 2018;30:22–5. 29504323

[28] Amodio P Pellegrini A Amistà P. Neuropsychological-neurophysiological alterations and brain atrophy in cirrhotic patients. Metab Brain Dis. 2003;18:63–78. 12603083 10.1023/a:1021982719654

